# Mitigation of Greenhouse Gas Emissions Through Straw Management and Oxygenated and Biochar-Based Fertilizers

**DOI:** 10.3390/plants14243791

**Published:** 2025-12-12

**Authors:** Qi Sun, Yu-Feng Wang, Hao Jiang, Huichang Bian, Xiao-Jun Wang, Yan Li, Hong-Sheng Gao, Xue Pan, Shuai Hao, Xue-Jia Gu

**Affiliations:** 1 Heilongjiang Academy of Black Soil Conservation & Utilization, Harbin 150086, China; 18800479543@163.com (Q.S.);; 2 Key Laboratory of Agro-Environment in Northeast Plain, Ministry of Agriculture and Rural Affairs, Harbin 150086, China; 3 School of Environment and Ecology, Jiangnan University, 1800 Lihu Avenue, Wuxi 214122, China

**Keywords:** straw return, greenhouse gases, rice, oxygenation measures

## Abstract

Straw returning is a common agricultural practice that can enhance rice (*Oryza sativa* L.) yield in paddy systems. However, it also leads to a significant increase in greenhouse gas emissions (GHG). Fortunately, this negative impact can be mitigated by implementing enhanced oxygenation strategies during rice cultivation. This study explored the effects of various oxygenation measures on GHG under straw-returning conditions through controlled pot experiments. Six distinct treatments were applied. These included straw not returned (NR, no straw applied), straw returned (SR), controlled irrigation (CI), oxygenation irrigation (OI), application of oxygenated fertilizer (OF, CaO_2_), and use of biochar-based fertilizer (CF). All treatment groups, with the exception of the NR group, involved the return of straw to the field. Creating rice production methods that increase yield and decrease emissions is of great importance to agricultural ecology. We postulated that using aeration methods under straw return conditions would stabilize rice yield and reduce GHG. The experimental results were consistent with our hypothesis. The experiment evaluated multiple parameters, including rice yield, leaf photosynthetic performance, soil ammonium and nitrate nitrogen (N) levels, and greenhouse gas emissions. The findings revealed that different oxygenation approaches significantly promoted rice tillering. Oxygenation measures have been shown to enhance rice yield by 19% to 65%. The highest tiller numbers were observed in the SR (22.75) and CF (21.6) treatments. Among all treatments, the CF achieved the highest seed setting rate at 0.94, which was notably greater than that of the other treatments. Total plant biomass was also significantly higher in the straw returning treatment (109.36 g), surpassing all other treatments. In terms of soil nitrogen dynamics, the OF treatment resulted in the highest nitrate nitrogen content. Meanwhile, the ammonium nitrogen concentrations across the four oxygenation treatments (CI, OI, OF, CF) ranged from approximately 7 to 8.9 mg kg^−1^. Regarding GHG, the CF treatment exhibited the lowest methane emissions, which were 33% lower compared to the straw returning treatment. The OF led to a 22% reduction in carbon dioxide emissions (CO_2_) relative to straw returning. Most notably, the CF reduced nitrous oxide emissions by 37% compared to the straw returning treatment. Overall, SR was found to substantially increase GHG. In contrast, all tested oxygenation measures—CI, OI, OF, and CF—were effective in suppressing GHG to varying degrees. Among these, the CF and OF demonstrated the most balanced and outstanding effects, both in reducing emissions and maintaining stable rice yields.

## 1. Introduction

China stands as the world’s foremost producer of rice (*Oryza sativa* L.), contributing approximately 28% of the global rice output. Data from the Food and Agriculture Organization (FAO) indicate that China’s rice planting area constitutes nearly one-fifth of the worldwide total [1]. Over the years, rice production has demonstrated a consistent upward trend, playing a pivotal role in safeguarding national food security and fostering socio-economic progress [2]. In parallel with the rise in crop yields, there has been a substantial increase in the generation of crop residues, particularly straw. Research shows that China produces over 800 million tons of crop straw annually, accounting for roughly 25% of the global straw output. Common methods for managing straw include returning it to the field, converting it into biochar, processing it into fuel or animal feed, and open-field burning [3]. Among these, returning straw to the field is widely regarded as an economical, efficient, and environmentally sustainable practice. It serves to replenish essential nutrients—such as nitrogen (N), phosphorus, potassium, and various micronutrients—that are vital for healthy crop growth. Moreover, this approach contributes to enhancing soil N balance, stimulating microbial activity, and influencing greenhouse gas emissions (GHG). At present, rapid global population growth has resulted in heightened levels of GHG, posing significant risks to both environmental sustainability and food security. Agriculture is a major contributor to these emissions, responsible for approximately 20–25% of the global total [4]. The primary greenhouse gases emitted from agricultural activities include carbon dioxide (CO_2_), methane (CH_4_), and nitrous oxide (N_2_O). Variations in the atmospheric concentrations of these gases exert a profound impact on the global climate system. It is estimated that over 13% of anthropogenic GHG—comprising around 60% of CH_4_ and N_2_O emissions—are linked to soil-derived gases and agricultural inputs [5].

While the practice of returning straw to the field can enhance soil fertility and boost crop productivity, it also presents certain adverse effects. These include potential negative impacts on early crop growth, increased GHG, and contributions to non-point source pollution [6]. As a water-intensive crop, rice cultivation under anaerobic (oxygen-deficient) conditions leads to substantial CH_4_ emissions. Studies reveal that cumulative CH_4_ emissions during the rice growing season account for as much as 91.2% of the annual total [7]. Within rice field soils, microorganisms metabolize organic matter in the straw through oxidation and reduction processes, generating reduced substances such as ferrous ions (Fe^2+^), manganese ions (Mn^2+^), and hydrogen sulfide (H_2_S). Research has indicated that Mn^2+^ can impair crop photosynthesis and suppress chlorophyll synthesis. Comparative studies have shown that straw return significantly elevates CH_4_ emissions, with increases of up to 73.52% compared to fields where straw is not returned [8]. The influence of straw returning on N_2_O emissions remains uncertain. While some investigations report that straw incorporation leads to increased N_2_O emissions [9], other studies—including work by Xia et al. [10]—suggest that long-term straw return can actually help reduce N_2_O emissions in paddy fields.

Farmland oxygenation technology was initially developed and introduced from overseas. This concept was put forward collaboratively by Professor David Midmore of the University of Queensland in Australia and Professor Su Ninghu from James Cook University [11]. Oxygenation treatments have been shown to markedly alleviate soil hypoxia, leading to increased crop yields and improved water use efficiency [12]. For instance, Rao et al. [13] explored the effects of oxygenation on cotton, finding that it enriched the soil microbial community structure. Similarly, Chen et al. [14] investigated the impact of oxygenation on pineapple, observing a doubling of root zone soil respiration and enhanced root physiology. Common methods of farmland oxygenation currently in use include mechanical oxygenation, chemical oxygen dissolution, Venturi air jet oxygenation, and micro/nano-oxygenation [15]. Among these, mechanical oxygenation is the most widely adopted, involving the injection of oxygen into water via mechanical devices such as air pumps. Chemical oxygen dissolution relies on reactive agents like calcium peroxide (CaO_2_) and hydrogen peroxide (H_2_O_2_) to elevate dissolved oxygen levels in water. Hu et al. [16]. reported that root-zone oxygenation significantly boosted rice yields, mainly by increasing the number of effective panicles. Building upon traditional flooded rice cultivation, several water-saving irrigation strategies have been developed. These include alternate wetting and drying (AWD) irrigation, intermittent irrigation, and mulched dryland cultivation. Such methods conserve irrigation water and contribute to reduced CH_4_ emissions from rice paddies [17]. The mechanism behind CH_4_ mitigation through water-saving irrigation lies in altering the irrigation regime, which enhances soil aeration and positively influences the composition of the soil microbial community as well as the soil redox potential [18]. AWD irrigation, for example, has been shown to improve water use efficiency by 5–30% during the rice season [19]. By facilitating the entry of atmospheric oxygen into the soil and elevating the soil redox potential, AWD reduces the abundance of methanogenic archaea while promoting methanotrophic bacteria, thus curtailing CH_4_ emissions [20]. Research indicates that AWD can reduce CH_4_ emissions by as much as 83% [21], primarily by disrupting the anaerobic conditions necessary for methanogen activity [22]. Stepniewski et al. [23] reported a 23.1% reduction in CH_4_ emissions under AWD. In contrast, conventional flooded rice systems maintain continuous water coverage, which helps buffer the soil against temperature extremes and drought stress [24,25]. However, prolonged flooding of rice fields results in poor soil aeration, impaired root respiration, diminished microbial activity, and the buildup of toxic compounds [26]. The application of CaO_2_—an oxygen-releasing agent—in chronically flooded fields can help counteract these negative effects. When CaO_2_ reacts with water, it forms calcium hydroxide, which raises soil pH and mitigates acid stress. Simultaneously, CaO_2_ decomposes to release hydrogen peroxide, which aids in the breakdown of soil organic matter and enhances nutrient availability. Studies have shown that compared to conventional fertilization, the use of CaO_2_ increased rice yields by 0.45 t ha^−1^ [27]. Yuan et al. [28] noted that CaO_2_ releases oxygen rapidly, with a single application remaining effective for approximately 7 to 12 days. By improving the soil oxygen environment and microbial community structure [29]—particularly increasing the abundance of methanotrophs—CaO_2_ facilitates the oxidation of CH_4_, thereby contributing to CH_4_ emission reductions [30,31].

Biochar, another soil amendment, has demonstrated strong potential in enhancing various physicochemical properties of soil [32]. It helps alleviate drought and salinity stress, degrades harmful substances, and increases the soil’s organic carbon content [33]. Peng et al. [34] found that biochar application increased the abundance of methane-oxidizing genes (pmoA) by 26.7% and methanogenic genes (mcrA) by 3.6% compared to fields without biochar. The porous structure of biochar enhances soil aeration and boosts dissolved oxygen levels, creating a favorable environment for methanotrophic microbes. Qin et al. [35] reported that biochar-treated fields exhibited a 27.53% reduction in GHG relative to fields receiving only basal fertilizer. Similarly, Li et al. [36] observed a 24.7% decrease in CH_4_ emissions from rice fields treated with biochar, attributing this effect to the disruption of the flooded soil’s anaerobic conditions, reduced water-soluble organic carbon, and enhanced carbon availability for methanotrophs.

Despite these advances, the complex interrelationships between various oxygenation techniques applied under straw-returning conditions, rice GHG, soil nutrient dynamics, total reducing substances, and microbial communities remain insufficiently understood. To address this knowledge gap, the current study employs an outdoor pot experiment approach. This study employs an outdoor pot experiment method with different aeration treatments to investigate the relationship between various aeration measures and GHG from rice under straw incorporation. Based on the above, we tested the following hypothesis: this integrated practice would create a more oxygenated soil environment, leading to a significant reduction in CH_4_ emissions compared to straw incorporation under continuous flooding. The ultimate goal is to establish a scientific foundation for the promotion of environmentally sustainable, green rice production practices.

## 2. Results

### 2.1. Rice Yield Under Different Oxygenation Measures

Oxygenation measures have been shown to enhance rice yield by 19% to 65% (Table 1). Among the various oxygenation strategies applied, different methods led to a notable increase in the tiller number of rice plants. Specifically, the treatments involving SR and the application of CF resulted in the highest tiller numbers, recorded at 22.75 and 21.6 tillers per plant, respectively. These values were significantly greater than those observed in the NR and standard SR control treatments. The overall ranking of treatments based on tiller number was as follows: SR > CF > OI > CI > OF > NR. The primary reason is that under our experimental conditions, the positive fertilization effect of straw return outweighed its negative impacts on the soil redox environment. The SR treatment provided a substantial and sustained release of nutrients (especially N, potassium, and micronutrients) as the straw decomposed throughout the growing season, which strongly promoted rice growth and yield formation. While the lack of oxygenation in SR likely created a suboptimal, somewhat reduced soil environment, the sheer abundance of available nutrients was sufficient to compensate for this stress and drive the highest biomass accumulation and grain yield. In contrast, oxygenation measures (CF, OI, etc.), while creating a more favorable root-zone oxygen environment, might have also accelerated the decomposition of soil organic matter (including the added straw), potentially leading to a relatively faster, but less synchronized nutrient release pattern that did not fully match the rice plant’s peak demand periods. This could explain why their yields, though significantly higher than NR, were lower than SR. Essentially, the SR treatment achieved maximum yield at a significant environmental cost (high CH_4_ emissions), while the oxygenation treatments, particularly CF, successfully struck a balance by substantially reducing emissions while maintaining a high yield level close to that of SR. No significant differences were detected in plant height across all treatments. In terms of panicle length, the CI treatment produced the longest panicles, measuring 24.44 cm, which was significantly longer than those observed in the straw removal treatment. Conversely, the OF treatment resulted in the shortest panicle length. Nevertheless, all other treatments exhibited panicle lengths that were greater than those of the straw removal treatment. Regarding seed setting rate, the CF treatment achieved the highest rate at 0.94, which was significantly superior to all other treatments. Additionally, SR, CI, OF, and OI all demonstrated seed setting rates that were significantly higher than the straw removal treatment, with increases of 7.23%, 12.05%, 9.64%, and 10.84%, respectively. No significant differences were found in single panicle grain weight among the treatments. However, the ranking based on this parameter was: OI > SR > CF > CI > NR > OF. For plant total weight, the straw returning to the field (SR) treatment yielded the heaviest plants, at 109.36 g, which was significantly greater than all other treatments. CI, OI, and CF also resulted in higher plant total weights compared to the OF treatment, and all were significantly higher than the straw removal treatment. In terms of thousand-grain weight, the SR treatment produced a value that was significantly higher than both the NR and CF treatments. No significant differences in thousand-grain weight were observed between the SR treatment and the other remaining treatments.

### 2.2. Photosynthetic Parameters of Rice Leaves Under Different Oxygenation Measures

**Figure 1:**
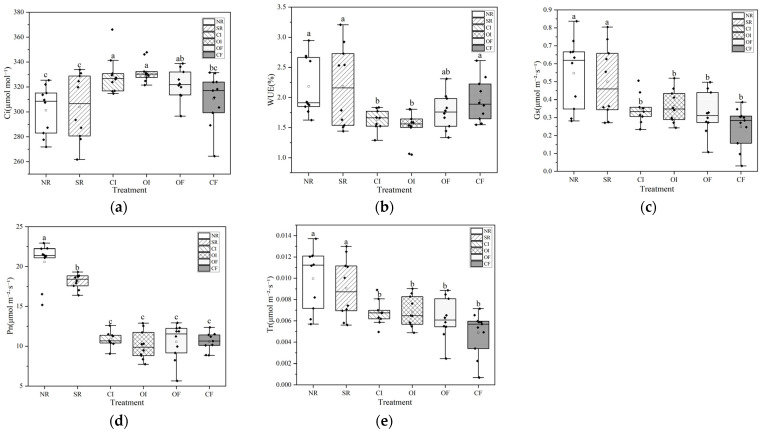
Photosynthetic parameters of rice leaves under different oxygenation measures. (**a**) Inter-cellular CO_2_ concentration, Ci; (**b**) Water use efficiency, WUE; (**c**) Stomatal conductance, Gs; (**d**) Net photosynthetic, Pn; (**e**) Transpiration rate, Tr; Different lowercase letters following the data (e.g., a, b, c) within the same column indicate significant differences among treatments according to a multiple comparison test (*p* < 0.05).

Overall, the CI and OI treatment groups exhibited the highest intercellular CO_2_ concentration (Ci) levels, with median values ranging from 325 to 330 µmol mol^−1^ (Figure 1). The data distribution within these groups was relatively concentrated, reflecting stable and consistent treatment effects. NR group followed closely, with a median Ci concentration of approximately 322 µmol mol^−1^. SR and CF groups were in the mid-range, with median Ci values between 306 and 322 µmol mol^−1^. Within this mid-range, the SR group displayed greater variability in the data, whereas the CF group showed a more tightly clustered distribution. NR group had the lowest median Ci concentration at 308 µmol mol^−1^. Notably, both the CI and OI groups contained high-value outliers that exceeded 340 µmol mol^−1^, indicating that certain plants under these treatments achieved substantially elevated Ci levels. Meanwhile, the SR group demonstrated the widest overall data range (approximately 261 to 367 µmol mol^−1^), signifying considerable variation in plant responses to this particular treatment. In summary, the CI and OI groups significantly enhanced intercellular CO_2_ concentration. In contrast, treatments involving NR and SR were associated with comparatively lower Ci concentrations. Among the water use efficiency (WUE) metrics, the SR group showed the most notable improvement, with a median WUE value of about 2.15. However, the data spread was relatively wide, with a box spanning from 1.5 to 2.6, and whiskers extending from 1.5 to 3.2. The NR group had a median WUE of approximately 1.9, with a box range of 1.8 to 2.6 and whiskers reaching from 1.6 to 2.9, including a few high outliers. The CF group recorded a median WUE of around 1.9 as well, with a box spanning 1.6 to 2.3 and whiskers from 1.5 to 2.6, indicating slightly greater dispersion compared to the NR and SR groups. The OI group exhibited the lowest WUE, characterized by the narrowest box, the smallest interquartile range (IQR), and highly concentrated data. Its median was about 1.6, with whiskers extending only from approximately 1.5 to 1.8 and no outliers present. Similarly, the CI group had highly concentrated data, with a median WUE of around 1.6, a narrow whisker range (about 1.3 to 1.8), and no obvious outliers. Regarding stomatal conductance across the different treatments, the order from highest to lowest was NR > SR > OI > CI > OF > CF. The NR treatment had the highest stomatal conductance, with a median of approximately 0.6 and a relatively dispersed data distribution. The SR group showed data patterns similar to those of the NR group. Overall, stomatal conductance values demonstrated a general stepwise decline from the SR group down to the CF group. The SR group’s values were second only to those of the NR group, while the OI and OF groups fell at intermediate levels, and the CF group recorded the lowest stomatal conductance. For net photosynthetic rate (Pn), the six treatment groups displayed a very clear and significant gradient decline. Moving from the NR group to the CF group, the positions of the boxes on the graph shifted noticeably downward, highlighting the substantial impact of the different treatments on the photosynthetic capacity of rice leaves. The NR group exhibited the highest net photosynthetic rate among all groups, with a median close to 22 μmol m^−2^ s^−1^ and a tightly clustered data distribution with minimal fluctuation. This suggests that rice leaves under the NR treatment maintained exceptionally high photosynthetic efficiency. The SR group had a lower net photosynthetic rate than the NR group but still higher than the subsequent four treatment groups. The CI, OI, and OF groups all had net photosynthetic rates at low and similar levels, with median values roughly around 10 μmol m^−2^ s^−1^. The differences among these three groups were much smaller compared to the gaps between them and the NR and SR groups. The CF group recorded the lowest net photosynthetic rate of all, with a median even lower than those of the CI, OI, and OF groups, indicating that this treatment most severely inhibited photosynthetic activity. As illustrated in Figure 1, the NR and SR groups also exhibited the highest transpiration rates among all six treatments, making them the two treatments associated with the most vigorous water transpiration. Within these, the NR group may have had a slightly higher median transpiration rate than the SR group, but both were at the uppermost level. The OI and OF groups had moderate transpiration rates, which were significantly lower than those of the NR and SR groups but noticeably higher than those of the CI group. The CI group had the lowest transpiration rate across all treatments, with a median that was far below the others, indicating that this treatment strongly suppressed water loss. The CF group also had a relatively low transpiration rate, though it may have been slightly higher than that of the CI group.

### 2.3. Nitrate and Ammonium N Content in Rice Soil Under Different Oxygenation Measures

**Figure 2:**
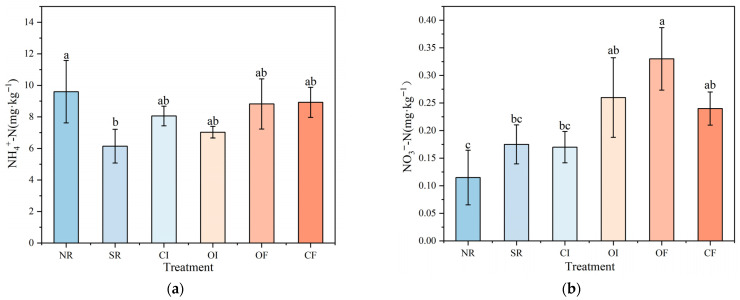
Nitrate and ammonium N content in rice soil under different oxygenation measures. The left figure (**a**) shows the ammonium N content in paddy soil under different oxygenation measures, and the right figure (**b**) shows the nitrate N content. Different lowercase letters for the same indicator indicate significant differences among treatments (*p* < 0.05).

The ammonium N content was highest in the NR group, reaching approximately 9.6 mg kg^−1^ (Figure 2). This suggests that under hypoxic or anaerobic soil conditions, ammonium N tends to accumulate more readily. In contrast, the SR group exhibited the lowest ammonium N content, at around 6.1 mg kg^−1^ (Table S1). Statistical analysis indicated a significant difference between the SR and NR groups (*p* < 0.05), with the SR group showing markedly lower ammonium N levels. The remaining four treatment groups—CI, OI, OF, and CF—had ammonium N contents ranging from 7 to 8.9 mg kg^−1^. Overall, the ranking of ammonium N content across all treatment groups was as follows: NR > CF > OF > CI > OI > SR.

For nitrate N, the OF group recorded the highest content, at approximately 0.33 mg kg^−1^, which was significantly higher than that observed in the CI, SR, and NR groups. There was no statistically significant difference between the OF group and the OI and CF groups, which had nitrate N contents of 0.26 mg kg^−1^ and 0.24 mg kg^−1^, respectively. The CI and SR groups both had slightly higher nitrate N levels than the NR group, although these differences were not statistically significant. The overall ranking of nitrate N content among the treatment groups was: OF > OI > CF > SR > CI > NR.

### 2.4. GHG from Rice Under Different Oxygenation Measures

**Figure 3:**
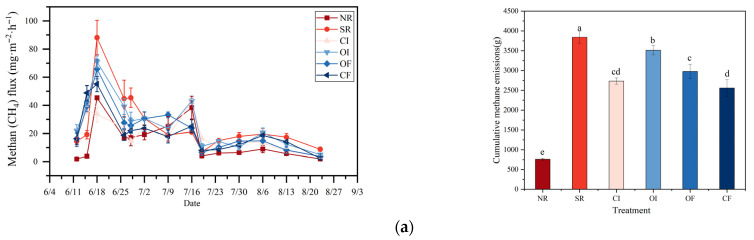

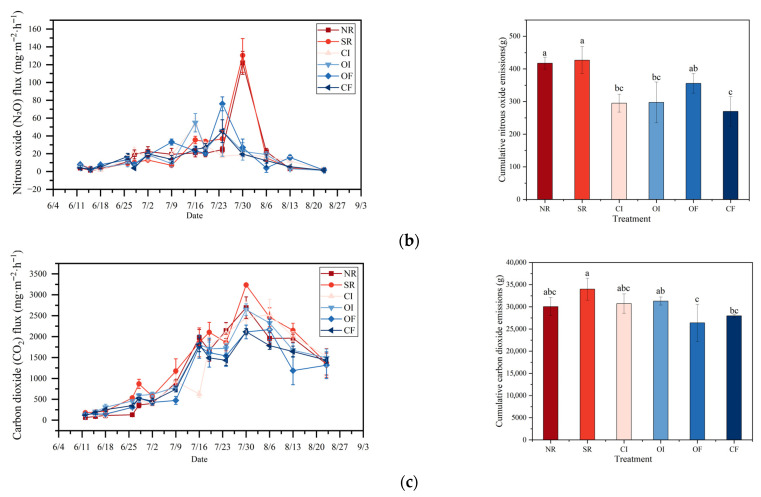
GHG from rice under different oxygenation measures. The line chart on the left shows the flux of GHG, while the bar chart on the right shows the cumulative GHG. (**a**) CH_4_ emission flux and cumulative emissions, (**b**) N_2_O emission flux and cumulative emissions, (**c**) CO_2_ emission flux and cumulative emissions. Different lowercase letters for the same indicator indicate significant differences among treatments (*p* < 0.05).

The cumulative CH_4_ emissions, ranked from highest to lowest, are as follows: SR >OI > CI > OF > CF > NR (Figure 3). Among these, SR exhibits significantly higher CH_4_ emissions compared to all other treatment groups, being approximately 400% greater than NR (Table S2). In contrast, NR shows the lowest CH_4_ emissions, which are significantly lower than those of all other treatments. OI produces CH_4_ emissions that are significantly higher than those observed under CI, OF, and CF, although they remain 29% lower than those under SR. OF results in higher CH_4_ emissions than CF, whereas no significant differences are found between CI and either OF or CF. Among all oxygenation-related treatments, CF demonstrates the lowest CH_4_ emissions, which are 33% lower than those from SR. However, an unusually sharp and pronounced CH_4_ flux peak occurred around June 18, during which the flux rose dramatically (Table S3). This spike is attributed to the temporary presence of methanogenic substrates, likely from residual organic matter. Since no additional exogenous carbon (such as straw) was supplied, the availability of methanogenic substrates quickly became limited, causing the CH_4_ flux peak to subside shortly thereafter. In contrast, the straw incorporation treatment maintained consistently high CH_4_ flux levels throughout the entire monitoring period, significantly and steadily exceeding the flux levels observed in all other treatments. This result confirms that under conventional flooded and incorporated management practices, the decomposition of straw leads to continuous and intense CH_4_ emissions.

Regarding cumulative CO_2_ emissions across all treatments, the ranking from highest to lowest is SR > OI > CI > NR > CF > OF. SR yields the highest CO_2_ emissions, which are significantly greater than those from OF and CF, and are also 13% higher than those from NR (Table S5). OI results in significantly higher CO_2_ emissions than OF, with no significant differences observed between NR, CI, OI, and CF. OF emits 22% less CO_2_ than SR, and CF emits 18% less than SR. Across all treatment groups, the CO_2_ flux exhibited a consistent temporal pattern: it initially rose, then subsequently declined. Flux levels were relatively low from early June to early July, began to increase significantly in mid-July, peaked during the mid-to-late July period, and then gradually declined from August through early September. This temporal trend closely corresponds to fluctuations in temperature and the progression of straw decomposition stages. Over the entire observation period, the treatment group without straw incorporation (NR) exhibited the lowest CO_2_ flux.

As a control treatment with no straw applied, the N_2_O emission flux in the no-straw (NR) group remained consistently low throughout the observation period (Table S4). During the early phase of the observation period (before mid-July), the OI treatment exhibited higher N_2_O emission fluxes than the CI treatment, and even displayed a secondary emission peak around July 30. However, in the later period, its emission levels were either similar to or slightly lower than those of the CI treatment. The CF and OF treatments emerged as the most effective in reducing N_2_O emissions, with flux levels remaining at extremely low levels throughout the entire period—comparable to or even lower than those in the NR treatment. For N_2_O emissions, the order from highest to lowest is SR > NR > OF > OI > CI > CF. Both NR and SR produce significantly higher N_2_O emissions compared to CI, OI, and CF. Among all treatments, CF shows the lowest N_2_O emissions, which are significantly lower than those from OF and 37% lower than those from SR. No significant differences in N_2_O emissions are detected between CI, OI, and CF.

## 3. Discussion

### 3.1. Effects of Different Oxygenation Measures on GHG from Rice

The general decrease in CH_4_ flux after the initial growth stage (observed in all treatments) is attributed to the development of root aerenchyma, which facilitates CH_4_ transport from the anaerobic soil to the atmosphere, and the gradual depletion of labile carbon sources [37]. The emission of the greenhouse gas CH_4_ is influenced not only by its production but also by processes such as oxidation. Paddy fields typically exhibit oxygen-deficient conditions, and introducing oxygenation treatments can enhance the oxidation of CH_4_, thereby reducing its overall emissions. The straw removal treatment demonstrated CH_4_ flux levels close to zero for the majority of the observation period [38]. The cumulative CH_4_ emissions from the OI and OF treatments were relatively similar, and both were lower than those from the SR treatment. While OI and OF treatments did improve local oxygen availability to some extent, their overall effectiveness in suppressing CH_4_ emissions was fairly limited over the full rice growing season. Their cumulative emissions were significantly lower than those observed in the SR, OI, and OF treatments. Meanwhile, the CF treatment exhibited CH_4_ suppression effects that were comparable to those of CI. This suggests that biochar, through its ability to improve soil structure, contributes to a sustained and beneficial effect in reducing cumulative CH_4_ emissions. The substantial reduction in CH_4_ emissions under OI and CI is linked to the introduction of oxygen during drying phases, which inhibits obligate anaerobic methanogens and promotes the activity of methane-oxidizing bacteria (methanotrophs) [39]. The transient spike in CH_4_ flux following CaO_2_ application is interpreted as a possible short-term stimulation of microbial activity due to the heat and pH change associated with peroxide decomposition [40], followed by suppression as oxygen is released.

Different oxygenation strategies produced markedly varied effects on CO_2_ flux, highlighting that the regulation of soil oxygen conditions can effectively influence the rate of straw decomposition and the intensity of CO_2_ emissions. In the absence of additional oxygenation and with no straw input, microbial activity and straw decomposition were comparatively weak, resulting in the lowest levels of CO_2_ emissions [41]. In contrast, the CF and OF treatments displayed very high CO_2_ flux peaks between early July and early August, far exceeding those of the other treatments. This suggests that the addition of fertilizer—particularly the biochar-based fertilizer—strongly stimulated soil microbial activity, accelerated the decomposition of straw, and consequently led to a sharp rise in CO_2_ emissions. The higher CO_2_ fluxes from SR and CF (Figure 1c) are interpreted as a result of enhanced microbial respiration fueled by added organic carbon [42]. The increase in CO_2_ flux towards the reproductive stage is linked to increased root exudation and autotrophic respiration [43], a pattern consistent with crop growth stages. The CO_2_ flux levels in the straw incorporation and controlled irrigation treatments remained at a moderate level throughout the observation period. These fluxes were higher than those in the NR treatment but significantly lower than those in the CF and OF treatments. These two treatments had a moderate stimulatory effect on straw decomposition. The oxygenated irrigation treatment showed a flux curve similar to that of the SR and CI treatments during the early phase, but displayed relatively higher flux values in the later phase (mid-to-late August). This may imply that direct oxygen injection had a more prolonged stimulatory impact on straw decomposition [44]. When compared to the NR, most oxygenation treatments led to increased CO_2_ flux, indicating that they generally enhanced straw decomposition. Among these, the addition of biochar-based fertilizer had the most pronounced stimulating effect on CO_2_ emissions. The effects of all treatments varied over time, with peak emissions occurring in July, likely due to optimal temperature and moisture conditions. For scenarios prioritizing rapid straw decomposition, the CF and OF treatments were highly effective. However, if the goal also includes controlling GHG, milder oxygenation approaches such as SR or CI may be more appropriate.

The limited input of easily decomposable, exogenous organic carbon resulted in low soil microbial activity and restricted denitrification processes, leading to N_2_O emissions that were much lower than those observed in the SR treatment. This outcome inversely demonstrates that the input of organic carbon serves as a key driver of N_2_O production [45]. The frequent oxygenation events in the early period likely created an environment of rapid “aerobic-anoxic” alternation, which is highly conducive to both nitrification (the conversion of ammonium N to nitrate N) and subsequent denitrification (the conversion of nitrate N to N_2_O and N_2_), thereby increasing N_2_O emissions [46]. Straw return creates a highly anaerobic environment, which inhibits nitrification and channels N transformation primarily through incomplete denitrification, leading to substantial N_2_O production and emission. Oxygenation measures improve the soil redox environment, creating favorable conditions for nitrification and shifting the N transformation pathway toward nitrification. Although nitrification may also produce a small amount of N_2_O, the net effect is a significant reduction in total N_2_O emissions because it greatly suppresses denitrification, the main source of N_2_O. This improves soil N status, retaining more N in the form of nitrate N and enhancing N use efficiency. These water management practices create ideal conditions for nitrification (during aerobic phases) and subsequent denitrification (upon re-flooding), which is a primary source of N_2_O [47]. The higher cumulative N_2_O emissions from OI and CI (Figure 1b, right) are mechanistically explained. These water management practices create ideal conditions for nitrification (during aerobic phases) and subsequent denitrification (upon re-flooding), which is a primary source of N_2_O [48]. The lower N_2_O emissions from CF are attributed to biochar’s capacity to adsorb N, thereby reducing substrate availability for nitrification/denitrification, and potentially promoting the complete denitrification of N_2_O to N_2_ [49].

In the later period, the extended OI weakened this stimulatory effect. The CI treatment maintained a relatively stable N_2_O emission flux throughout the observation period, at a medium level. The CI inherent to this treatment also create an environment of alternating oxidation and reduction, although the frequency and intensity of these fluctuations may not have been as pronounced as in the OI treatment, resulting in a comparatively milder stimulation of N_2_O emissions. Biochar’s porous structure significantly enhances soil aeration, thereby helping to prevent the formation of strongly anaerobic microenvironments [50]. Additionally, biochar can adsorb N and organic molecules, slowing their microbial utilization and thus inhibiting denitrification. It may also promote more complete denitrification pathways, favoring the production of N_2_ over N_2_O. CaO_2_, used in the OF treatment, slowly releases oxygen upon contact with water, helping to maintain an oxidative state in the root zone soil over an extended period. This strongly suppresses denitrification and effectively reduces N_2_O generation.

### 3.2. Effects of Different Oxygenation Measures on Rice Yield and Its Components

The SR treatment exhibited the best performance in terms of tiller number, indicating the potential for achieving the highest effective panicle density per unit area—an essential factor contributing to its high grain yield. In addition, SR also produced rice grains with the highest 1000-grain weight, suggesting plumper and potentially higher-quality grains. A higher grain weight typically indicates better grain filling, leading to larger and denser kernels. This improved physical characteristic is a key indicator of superior grain quality [51]. The application of straw returning enriched the soil with substantial amounts of organic matter and essential nutrients, thereby comprehensively promoting overall rice growth. This makes straw returning the most direct and effective agronomic measure for increasing rice yield [52]. The CF treatment demonstrated its primary advantage in having the highest spikelet fertility rate. It is likely that biochar contributed to improved soil structure and enhanced water and nutrient retention properties, creating a more stable microenvironment for grain filling. This likely reduced the occurrence of unfilled grains and thereby increased the spikelet fertility rate. Furthermore, the tiller number under CF was also relatively high, ranking second only to that observed in the SR treatment [53]. The OI and CI water management strategies optimized the oxygen availability in the rhizosphere through practices such as CI and OI. These methods enhanced root vigor, which translated into better performance in terms of panicle length and spikelet fertility rate, ultimately resulting in considerable grain yields [54]. In contrast, the OF treatment did not exhibit outstanding performance across multiple growth indicators, such as plant height and panicle length. This may be attributed to certain chemical effects—such as alterations in soil pH—caused by calcium peroxide during the oxygenation process, which could have slightly inhibited rice growth [55]. As the control treatment, the NR approach, which involved no application of straw, failed to replenish soil fertility. Consequently, it resulted in generally lower values for key yield-related indicators, including tiller number, spikelet fertility rate, and 1000-grain weight, ultimately leading to the lowest final grain yield among all treatments. This outcome inversely underscores the critical importance of supplying exogenous organic carbon to sustain high rice productivity. Among all treatments, SR emerged as the optimal strategy for increasing rice yield. It directly and effectively enhanced nearly all major yield components—including tiller number, panicle development, and grain weight—by enriching the soil with organic matter and essential nutrients [56]. Oxygen is the primary energy source required for the normal metabolic activities of oxygen-demanding cells, particularly those in crop roots. Restricted soil porosity can confine roots in an environment of prolonged oxygen deficiency. Oxygenation-based treatments, such as OI and CI, facilitate the delivery of oxygen to the soil in close proximity to crop roots. This increases the oxygen supply to root tissues, ensures optimal root functionality, stimulates the activity of beneficial soil microbes, and promotes efficient mineral transformation within the rhizosphere. These combined effects contribute to enhanced crop growth and yield. Synthesizing findings from previous studies on GHG, the CF treatment demonstrates notable advantages. While maintaining high grain yields, it achieves the highest spikelet fertility rate and concurrently minimizes emissions of CH_4_ and N_2_O to the greatest extent. Both CI and OI treatments also show significant yield-increasing benefits and outperform simpler oxygenation-based approaches like OF. Therefore, if the primary objective is to achieve the highest possible grain yield, SR is the most effective choice. However, if the goal shifts toward promoting green and sustainable agriculture—ensuring relatively high yields while simultaneously achieving significant reductions in GHG—then CF represents the most comprehensive and advantageous solution.

### 3.3. Effects of Different Oxygenation Measures on Rice Photosynthesis Parameters

The median values of net Pn and Gs in the NR and SR treatments were generally lower compared to those observed in the OF, OI, and CF treatments. The SR treatment may have intensified soil hypoxia and generated certain inhibitory compounds during the early stages of straw decomposition, both of which negatively impacted root and leaf physiological functions [41]. The gradual decomposition of straw may influence soil structure and create localized oxygenated zones, enhancing root aerobic respiration and nutrient uptake efficiency, as better N nutrition supports chlorophyll synthesis [57]. These findings suggest that straw returning alone, in the absence of effective oxygenation strategies, could be detrimental to plant photosynthesis [58]. Alternate wetting and drying cycles regulate phytohormone balance, leading to optimized stomatal behavior and carbon assimilation. The improved WUE under these treatments is linked to their role in moderating transpirational Tr while maintaining CO_2_ diffusion [59]. In contrast, the OF treatment exhibited the highest median values of both Pn and Gs, indicating its superior ability to fix CO_2_ per unit leaf area and the greatest degree of stomatal opening, which ensured an ample supply of CO_2_ for photosynthetic processes. The application of CaO_2_ facilitated a slow and steady release of oxygen, effectively alleviating hypoxic conditions in the root zone [60]. This enhancement in root zone oxygenation promoted root vitality and improved nutrient uptake, which in turn triggered signaling mechanisms that led to greater stomatal opening in the leaves and supported more efficient photosynthesis [61]. The immediate oxygen release from CaO_2_ decomposition in relation to its transient but significant effect on root zone oxygenation [62], particularly benefiting plants during critical growth stages when soil oxygen demand peaks. The context of biochar’s dual role in improving soil porosity while potentially inducing temporary N immobilization [63], creating a complex interplay that affects photosynthetic. The CF treatment demonstrated a balanced performance in terms of carbon assimilation and water conservation. It showed a significantly higher WUE than all other treatments, meaning that rice leaves under the CF treatment were able to fix more CO_2_ per unit of water transpired [64]. Biochar contributed to establishing a stable growing environment for plants. The median values of both Gs and Pn under the biochar treatment were relatively high, ranking just below those of the OF treatment. Although regular oxygenation of irrigation water directly elevated oxygen levels in the rhizosphere—producing effects similar to those of the OF treatment—its overall stability may be marginally lower than that of the OF treatment, which provides a continuous and sustained release of oxygen.

### 3.4. Effects of Different Oxygenation Measures on Nitrate and Ammonium N Content in Rice Soil

The ammonium N content was highest in the NR treatment. This outcome can be attributed to the absence of straw, which serves as an important carbon source for soil microorganisms. Without this carbon input, microbial activity was significantly reduced, leading to weaker retention of ammonium N in the soil. Furthermore, the relatively poor aeration conditions in the NR treatment likely hindered the nitrification process—the biological conversion of ammonium N to nitrate N—resulting in the accumulation of ammonium N. In contrast, the SR treatment exhibited the lowest ammonium N content. The presence of straw provided an abundant and readily available carbon source, which substantially stimulated microbial activity. Microorganisms actively assimilated large quantities of ammonium N for their own growth and metabolic processes. This biological uptake effectively lowered the level of plant-available (or ineffective) N in the soil [65]. However, this also implies that the SR treatment demands careful N management to prevent potential N deficiencies in crops, as much of the N is temporarily immobilized by microbes. Among all oxygenation treatments, the ammonium N content fell between that observed in the NR and SR treatments, with no statistically significant differences among the oxygenation treatments themselves. This suggests that oxygenation practices established a balanced effect: on one side, enhanced aeration facilitated nitrification, leading to the consumption of a portion of ammonium N; on the other side, the improved soil environment may have concurrently accelerated the mineralization of organic N, thereby releasing additional ammonium N into the soil system [66,67].

In terms of nitrate N content, the OF and CF treatments yielded the highest levels in the soil. These two treatments were the most effective in promoting nitrification. In the case of OF, CaO_2_ acted as a steady oxygen source, continuously releasing oxygen into the soil and establishing highly favorable conditions for nitrifying bacteria, which are strictly aerobic microorganisms. This environment enabled the rapid conversion of ammonium N into nitrate N. Similarly, CF enhanced soil structure by improving pore connectivity and created a stable, well-aerated environment over the long term, which also significantly boosted the nitrification process. CI and OI also contributed to conditions that were conducive to nitrification, although their effects were comparatively less pronounced and consistent compared to those of OF and CF. In the SR treatment, despite the overall reduction in available N due to microbial retention, the prevalence of anaerobic conditions under SR likely led to the loss of nitrate N through gaseous denitrification pathways, such as the emission of N_2_O or N_2_. Moreover, the NR treatment was not only susceptible to potential denitrification losses but may have also started with a lower total N content due to its inherently poorer soil fertility status.

### 3.5. Economic and Social Benefits of Oxygenation Measures for Rice Cultivation

By improving the soil environment and nutrient availability, oxygenation measures can help maintain or even increase rice yields, directly boosting farmers’ income. Oxygenation measures can maintain or even enhance the stability of grain yields. Significantly reducing GHG from rice paddies helps mitigate climate change, fulfilling agriculture’s role in environmental conservation. The significant reduction in GHG could translate into economic benefits under future carbon pricing or agricultural carbon sequestration policies. It aligns with global “green development” initiatives, such as China’s “Peak Carbon and Carbon Neutrality” goals, and may qualify for government ecological compensation or subsidies. Improve soil conditions can increase nutrient utilization efficiency, potentially reducing fertilizer application rates and input costs over the long term. The potential for improving soil health (e.g., through the long-term persistence of biochar in the soil) may reduce future fertilizer inputs and enhance resilience to climate stress, representing a long-term investment. Currently, the production costs of biochar and oxygenated fertilizers are higher than those of conventional fertilizers. However, with technological advancements, scaled-up production, and potential government subsidies for green agriculture, these costs are expected to decrease significantly. Evaluating the value-to-cost ratio through long-term, large-scale field trials integrated with economic analysis is a critical next step toward the practical application of these promising fertilizers.

### 3.6. Limitations

In this section, we acknowledge that pot experiments, by their nature, can alter soil microenvironments (e.g., root confinement, limited soil volume, and controlled water regime), which can in turn affect plant growth, nutrient dynamics, and greenhouse gas emissions. These factors may indeed influence the scalability of our findings. We have explicitly stated that our results should be interpreted as proof-of-concept under controlled conditions, and that their direct extrapolation to field settings requires validation through future large-scale field studies. However, we also highlight that the primary value of this pot experiment was to isolate and identify the specific mechanisms (e.g., the interplay between aeration, straw decomposition, and N transformations) in a way that is difficult to achieve in complex field environments. This controlled approach provides a critical foundation for designing targeted field experiments.

## 4. Materials and Methods

### 4.1. Site Location, Soil Analysis and Current Agricultural Practice

**Figure 4:**
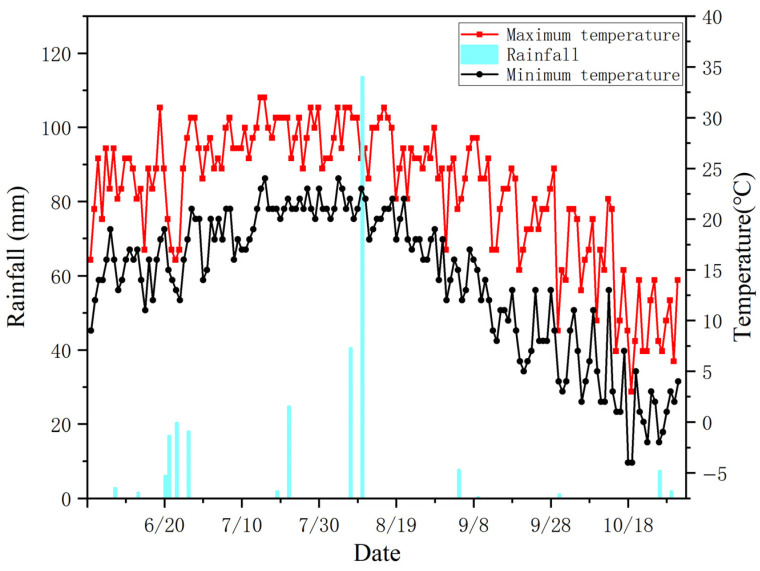
Temperature changes and rainfall at the rice growth period test site in 2024. The red line represents the highest temperatures, the black line represents the lowest temperatures, and the blue bar chart represents the rainfall.

The experiment was carried out between June and October 2024 at the potting field of the Heilongjiang Academy of Agricultural Sciences, located at latitude 45.68° N and longitude 126.61° E. The region experiences a continental monsoon climate. In Harbin, where the experimental site is situated, the annual average precipitation is 367 mm, and the typical diurnal temperature variation is approximately 12 °C. The specific patterns of temperature and rainfall recorded at the experimental location during 2024 are illustrated in Figure 4.

The test soil used in the experiment was classified as black soil, and its fundamental physical and chemical properties are detailed in Table 2. The soil samples were collected from Suihua City, Heilongjiang Province. After collection, the soil was stored in a cool location to allow for natural air-drying, and subsequently passed through a 5.0 mm sieve to ensure uniform particle size. The sieved soil was thoroughly mixed to achieve homogeneity, and a total of 11 kg of the processed soil was placed into each polyethylene pot. Each pot had a soil layer with a height of 34 cm and a diameter of 27 cm. The rice variety cultivated in 2024 across all experimental pots was Suijing 18. In Heilongjiang Province, most rice growers follow a conventional spring land preparation approach. Specifically, after the autumn harvest, rice straw is typically chopped into small pieces and evenly distributed back into the field. In the subsequent spring season, base fertilizer is applied to the soil, followed by dry rotary tillage to incorporate both the fertilizer and the straw into the soil layer. The field is then flooded, and the soil-water mixture is stirred to prepare for planting. For the purposes of this study, the straw was naturally air-dried and uniformly cut into 3 cm segments. These straw segments were then mixed with the soil and the base fertilizer prior to flooding the experimental fields. The nutritional composition of the straw included a N content of 0.69%, a phosphorus content of 0.17%, a potassium content of 1.78%, and a carbon content of 70.02%.

### 4.2. Trial Design

In the pot experiment, six distinct treatment groups were established to evaluate various agronomic practices: (1) NR—no straw was applied, and all straw was removed from the experimental units; (2) SR—straw was returned to the field at a uniform application rate; (3) CI—irrigation was managed through an alternating wetting and drying regime, with each irrigation event delivering a water depth of 8–10 cm, followed by natural drying until fine cracks became visible on the soil surface, after which rewatering was performed to the same depth; (4) OI—the irrigation water was aerated every three days during the first 20 days after rice transplanting, with the aeration frequency subsequently adjusted to approximately every five days, and each aeration session lasted for two hours [68]; (5) OF—calcium peroxide was applied at a rate of 0.57 g per pot (120 kg ha^−1^) each time fertilizer was administered, thereby introducing oxygen into the root zone through the fertilizer application [69]; and (6) CF—biochar was incorporated into the soil as a base fertilizer at a rate of 6.36 g per pot (900 kg ha^−1^), the biochar used in this experiment was produced from rice straw under anaerobic conditions at 550 °C, with a carbon content of 444.15 g kg^−1^ and a N content of 4.18 g kg^−1^). The diameter of the pot is 30 cm. All treatment groups, with the exception of the NR group, involved the return of straw to the field at a standardized rate of 48 g per pot (6800 kg ha^−1^, air-dry weight, approximately 10% moisture content). The straw was air-dried indoors to a constant weight before application, with a moisture content of approximately 10%. In Heilongjiang Province, rice growers mostly adopt spring land preparation methods, with straw being crushed and evenly spread in full amount back into the fields after the autumn harvest [70]. The basal fertilizer applied across treatments consisted of 2.03 g of urea (287 kg ha^−1^), 1.84 g of calcium superphosphate (260 kg ha^−1^), and 2.12 g of potassium chloride per pot (300 kg ha^−1^). The tillering fertilizer consisted of 1.52 g of urea per pot (215 kg ha^−1^), applied 10–15 days after transplanting, while the spike fertilizer, also 1.52 g of urea per pot (215 kg ha^−1^), was applied when approximately 80% of the second-to-last leaves on the main stems exhibited tip emergence. Each of the six treatment groups was replicated six times, and the experimental layout followed a randomized block design to minimize variability. Rice seeds were sown on May 10 and transplanted on June 10. Prior to transplanting, the pots were irrigated and allowed to stand for 24 h, after which a 3 cm water layer was added. The soil surface was manually leveled, and this water layer was maintained for seven days to facilitate transplanting. Each pot contained three hills, with three rice seedlings planted per hill.

The tillering fertilizer was applied 10–15 days after transplanting, and the spike fertilizer was applied when about 80% of the penultimate leaves on the main stems showed tip emergence. Before each fertilizer application, the existing water in the pot was carefully drained and retained. A small pit, 3–5 cm deep and positioned approximately 2 cm from the plant roots, was dug, into which the fertilizer was placed. The surrounding soil was then smoothed, and the pot was rinsed three times with the previously saved water to prevent fertilizer loss. Finally, 3–5 cm of water was added back to the pot to restore the appropriate water layer.

### 4.3. Sampling and Analysis

At maturity, soil samples were collected from the root zone (0–20 cm depth) of each pot. Fresh soil samples were immediately sieved (2 mm mesh) and extracted with a 2 M KCl solution (soil-to-solution ratio of 1:5) by shaking for 1 h. The extracts were then filtered. The concentrations of nitrate N and ammonium N in the filtrate were determined using a continuous flow analyzer. All concentrations are expressed on a dry soil weight basis (mg kg^−1^). Heading-flowering stage is a critical period for determining final grain yield. During this stage, the plant’s photosynthetic activity is at its peak, directly supporting grain filling. Measuring these key physiological parameters at this specific stage allows us to most effectively evaluate the impact of our experimental treatments on the core physiological processes governing rice productivity. At maturity, soil samples were collected from the root zone (0–20 cm depth) of each pot. For greenhouse gas collection and analysis, a closed static chamber method was employed. Sampling was carried out once per week throughout the rice growth cycle. Each sampling event was conducted between 9:00 and 10:00 AM, with gas samples collected at 0, 10, 20, and 30 min after the chamber was sealed [71,72]. At each time point, 45 mL of gas was extracted, and subsequent analysis of the gas samples was performed using gas chromatography. The CH_4_ and N_2_O fluxes were calculated according to the following equation [73].

(1) F=ρ×h×dc/dt×273/(273+T)

where *F* is the gas emission flux, *ρ* is the density of the gas under standard conditions (0.714 kg m^−3^ for CH_4_ and 1.964 kg m^−3^ for N_2_O), *h* is the height of the chamber (m), *T* is the average temperature inside the chamber (°C) during gas collection, and *dc*/*dt* is the change rate of gas concentration inside the chamber per unit time (ppm min^−1^). Cumulative emissions were calculated as follows:

(2) $$CE=\Sigma [(F_{i}+F_{i+1})/2\times 24\times d]$$

where *CE* is the cumulative gas emissions, *F_i_* and *F_i_*_+1_ are the gas emission fluxes at two consecutive adjacent sampling time points, and *d* is the number of days for the interval.

Photosynthesis parameters were measured using a Portable Photosynthesis System (model LI-6800, manufactured by LI-COR, Lincoln, NE, USA). The parameters assessed included net photosynthetic rate (Pn), stomatal conductance (Gs), transpiration rate (Tr), and intercellular CO_2_ concentration (Ci). Water use efficiency (WUE) was calculated as the ratio of net photosynthetic rate to transpiration rate (WUE = Pn/Tr), and the stomatal limitation value (Ls) was derived from the formula [1 − (Ci/Ca)], where Ca represents the atmospheric CO_2_ concentration. The leaf area exposed during measurements was 3 cm^2^, the atmospheric temperature was maintained at 28 °C, and the atmospheric CO_2_ concentration (Ca) ranged from 378.67 to 398.41 μL L^−1^.

All yield-related parameters were measured at the maturity stage of rice. The specific measurement methods are as follows: Height (cm): Measured from the soil surface to the tip of the main stem panicle at the mature stage; Number of tillers: The total number of tillers per plant was counted at maturity; Panicle length (cm): It was measured as the length of the main stem panicle from its base to the apex; “Single spike grain weight” refers to the air-dried grain weight of a single main panicle; Seed setting rate (%): It was calculated as the percentage of filled grains relative to the total number of grains on the main panicle. This measurement was conducted at physiological maturity. Specifically, grains that sank in water were considered filled, while those that floated were considered unfilled. The formula used was: Seed setting rate (%) = (Number of filled grains/Total number of grains) × 100%; Single panicle grain weight (g): The air-dried weight of grains harvested from the main panicle; Total Weight (g/pot): This represents the total air-dried weight of all grains harvested from each experimental pot; Thousand seed weight (g): Calculated by weighing 1000 filled, air-dried grains randomly sampled from the total yield of each pot.

### 4.4. Statistics

This study employed Microsoft Excel to conduct basic statistical analyses of all collected data. For more advanced variance analysis, IBM SPSS Statistics 25 was utilized, while Duncan’s multiple range test was applied to assess the significance of differences among group means. Finally, Origin 2024 was used to create the graphical representations of the data.

## 5. Conclusions

In conclusion, our findings robustly support the hypothesis that optimizing rhizosphere oxygen availability through targeted practices can effectively decouple the typical trade-off between methane (CH_4_) mitigation and yield maintenance in rice paddies under straw return (SR). The key mechanism identified is a shift in the soil N paradigm, where oxygenation measures, particularly continuous flooding (CF), promoted a more plant-available nitrate-dominated environment over ammonium, creating a stronger foundation for yield formation. Among the strategies tested, CF and organic fertilizer application (OF) emerged as the most effective. CF was exceptional in establishing a synergistic balance, achieving significant reductions in greenhouse gas emissions (particularly CH_4_) while securing the second-highest yield, supported by superior yield components. Although sole SR produced the highest yield, it came with a substantial emissions cost, which these oxygenation strategies successfully mitigated. Therefore, this study demonstrates that integrating straw return with oxygenation management—especially CF—presents a viable pathway toward achieving both agricultural productivity and environmental sustainability in paddy fields.

## Figures and Tables

**Table 1:** Rice yield and its components under different oxygenation measures. The data presented in this table are expressed as mean ± standard error (mean ± SE). Different lowercase letters following the data (e.g., a, b, c) within the same column indicate significant differences among treatments according to a multiple comparison test (*p* < 0.05).

	Number of Tillers	Height (cm)	Panicle Length (cm)	Seed Setting Rate (%)	Single Spike Grain Weight (g)	Total Weight (g/pot)	Thousand Seed Weight (g)
NR	15.43 ± 2.23 c	124.57 ± 5.14 a	23.00 ± 1.26 bc	0.83 ± 0.06 c	2.64 ± 0.91 a	66.04 ± 10.56 c	24.65 ± 1.18 b
SR	22.75 ± 3.06 a	128.81 ± 5.27 a	24.06 ± 1.12 ab	0.89 ± 0.02 b	3.03 ± 0.44 a	109.36 ± 9.15 a	26.92 ± 1.23 a
CI	18.80 ± 3.97 ab	129.29 ± 7.08 a	24.44 ± 1.08 a	0.93 ± 0.06 b	2.78 ± 0.30 a	87.02 ± 13.88 b	26.14 ± 0.25 ab
OI	19.50 ± 3.84 ab	126.55 ± 4.00 a	23.56 ± 0.68 abc	0.91 ± 0.04 b	3.07 ± 0.53 a	85.77 ± 22.77 b	25.20 ± 0.35 ab
OF	18.78 ± 4.27 ab	122.70 ± 4.30 a	22.67 ± 0.88 c	0.92 ± 0.04 b	2.62 ± 0.41 a	78.99 ± 19.09 bc	25.39 ± 1.46 ab
CF	21.60 ± 3.21 a	122.81 ± 9.62 a	23.10 ± 1.67 bc	0.94 ± 0.04 a	2.84 ± 0.59 a	90.43 ± 5.19 b	24.75 ± 0.43 b

**Table 2:** Basic physical and chemical properties of soil.

TN %	TP %	TK %	AN mg kg^−1^	AP mg kg^−1^	AK mg kg^−1^	OM g kg^−1^	pH
0.123	0.075	2.36	79.3	77.3	231	18.3	5.36

## Data Availability

The original contributions presented in the study are included in the article/Supplementary Material, further inquiries can be directed to the corresponding author.

## Supplementary Materials

The following supporting information can be downloaded at: https://www.mdpi.com/article/10.3390/plants14243791/s1, Table S1: Nitrate and ammonium nitrogen content in rice soil under different oxygenation measures. Table S2: Cumulative greenhouse gas emissions from rice under different oxygenation measures. Table S3: The flux of CH_4_ from rice under different oxygenation measures. Table S4: The flux of N_2_O from rice under different oxygenation measures. Table S5: The flux of CO_2_ from rice under different oxygenation measures.

## Abbreviations

The following abbreviations are used in this manuscript:

CO_2_	Carbon dioxide
N_2_O	Nitrous oxide
CH_4_	Methane
Pn	Net photosynthetic
Gs	Stomatal conductance
Tr	Transpiration rate
Ci	Inter-cellular CO_2_ concentration
WUE	Water use efficiency
CaO_2_	Calcium peroxide
N	Nitrogen
O_2_	Oxygen
