# Supplementary material for: Mitigation of Greenhouse Gas Emissions Through Straw Management and Oxygenated and Biochar-Based Fertilizers
Source: Plants (Basel). 2025 Dec 12;14(24):3791. doi: 10.3390/plants14243791 (PMC12736486; doi:10.3390/plants14243791)
Supplement: Supplementary file 1 [file plants-14-03791-s001.zip › plants-3984128-supplementary.pdf]

Supporting Information

FIVE TABLES

List of contents

Tables:

Table S1 Nitrate and ammonium nitrogen content in rice soil under different oxygenation measures..3
Table S2 Cumulative greenhouse gas emissions from rice under different oxygenation measures.
....4
Table S3 The flux of CH4 from rice under different oxygenation measures....................................5
Table S4 The flux of N_2O from rice under different oxygenation measures................................6
Table S5 The flux of CO_2 from rice under different oxygenation measures....................................7

Table S1 Nitrate and ammonium nitrogen content in rice soil under different oxygenation measures.

Treatment	NO_3^--N(mg/kg）	NH_4^+-N（mg/kg）
NR	0.12±0.05a	9.6±1.98c
SR	0.18±0.04b	6.15±1.07bc
CI	0.17±0.03ab	8.06±0.62bc
OI	0.26±0.07ab	7.03±0.37ab
OF	0.33±0.06ab	8.83±1.59a
CF	0.24±0.03ab	8.93±0.95ab

Table S2 Cumulative greenhouse gas emissions from rice under different oxygenation measures.

Treatment	CH_4 Emissions (g)	CO_2 Emissions (g)	N_2O Emissions (g)
NR	762.52±24.86e	30045.51±2057.74a	417.53±18.48abc
SR	3838.22±157.05a	33981.60±2476.30a	426.93±41.38a
CI	2734.69±79.89cd	30726.15±2197.06bc	295.13±27.52abc
OI	3513.41±116.50b	31302.86±922.82c	297.50±62.10ab
OF	2976.50±178.22c	26380.40±4181.25ab	355.67±29.82c
CF	2558.42±214.23d	27962.14±393.36c	269.79±45.40bc

Table S3 The flux of CH_4 from rice under different oxygenation measures.

Treatment / Date	NR	SR	CI	OI	OF	CF
6/12	1.88±0.03	14.46±3.05	16.82±5.58	22.06±4.19	16.38±5.67	16.35±4.01
6/15	3.90±0.13	19.20±3.00	36.53±2.77	41.69±1.45	39.30377±3.82	48.88±5.21
6/18	45.39±1.26	88.18±12.21	33.79±5.66	70.98±4.23	65.82±6.96	55.16±5.40
6/26	16.73±0.30	44.73±13.16	23.63±2.10	39.16±5.61	27.73±4.20	18.86±3.72
6/28	17.17±5.89	45.46±6.93	14.73±0.30	28.77±6.42	25.62±5.32	21.99±1.26
7/2	19.23±3.69	30.74±4.49	23.59±1.60	30.74±1.12	30.56±4.95	23.66±2.06
7/9	25.19±6.81	19.04±4.39	18.21±2.32	23.32±2.88	33.26±2.11	17.89±4.60
7/16	38.40±8.11	21.07±0.69	42.54±1.51	43.11±1.71	23.83±2.21	25.20±4.50
7/19	4.06±0.49	6.95±0.34	16.39±0.74	11.00±1.80	6.65±2.43	8.04±0.81
7/24	6.10±0.55	14.96±0.91	10.63±0.73	14.24±1.61	10.12±1.35	8.62±0.43
7/30	6.44±0.42	17.98±2.71	14.19±2.18	10.31±1.48	14.63±1.10	11.93±0.83
8/6	8.97±2.59	19.68±4.01	13.31±2.20	20.70±3.22	14.76±0.75	18.71±0.93
8/13	5.69±0.89	17.41±2.65	7.66±1.68	12.40±2.58	8.28±2.28	14.21±0.65
8/23	1.95±0.88	8.83±1.45	3.59±1.08	4.98±0.87	3.68±0.25	2.43±0.96

Table S4 The flux of N_2O from rice under different oxygenation measures.

Treatment / Date	NR	SR	CI	OI	OF	CF
6/12	3.92±1.15	7.89±1.22	7.30±1.28	4.41±0.73	8.01±1.16	4.04±0.41
6/15	1.54±0.50	3.43±0.49	2.74±0.46	2.40±0.39	2.36±0.90	2.74±0.32
6/18	2.28±0.50	3.44±1.08	1.58±0.22	4.33±2.28	7.74±0.46	5.02±1.16
6/26	11.13±2.81	9.15±2.21	14.15±3.90	8.85±3.44	13.39±2.57	16.94±3.48
6/28	19.23±4.99	10.09±2.39	22.83±4.52	8.75±1.10	8.04±1.86	3.92±1.29
7/2	22.49±5.42	12.98±1.57	15.54±3.67	18.88±4.31	17.81±3.30	19.88±3.83
7/9	19.33±6.63	6.99±0.20	18.42±1.63	8.85±3.59	33.12±3.40	13.74±4.3
7/16	20.99±4.42	35.49±4.18	52.77±7.15	54.89±10.36	22.95±3.53	25.07±3.54
7/19	20.54±3.89	33.76±2.26	29.74±3.22	24.73±0.64	20.70±2.95	26.74±4.68
7/24	24.61±3.55	36.78±9.02	17.25±0.44	45.67±28.54	76.08±7.81	45.35±12.75
7/30	122.05±12.87	130.47±18.99	18.76±2.94	22.64±9.76	26.98±9.53	19.56±1.1
8/6	22.27±3.70	15.30±3.56	16.72±2.53	19.06±3.21	4.11±4.98	12.3±7.22
8/13	3.21±0.48	4.11±0.46	12.07±4.78	3.08±0.53	15.91±2.53	5.48±0.47
8/23	1.62±0.39	1.31±0.08	1.03±0.11	1.22±0.27	1.57±0.39	1.38±0.21

Table S5 The flux of CO_2 from rice under different oxygenation measures.

Treatment / Date	NR	SR	CI	OI	OF	CF
6/12	64.35±4.18	64.35±4.18	141.95±21.8	103.06±14.79	146.26±11.02	119.35±8.27
6/15	89.68±25.9	89.68±25.9	133.13±21.97	207.01±32.24	147.59±7.46	187.13±9.35
6/18	115.01±55.17	115.01±55.17	124.31±53.04	310.95±73.31	148.93±19.58	254.91±25.37
6/26	130.29±13.6	130.29±13.6	308.88±60.04	461.94±29.57	308.7±37.9	350.01±8.42
6/28	364.21±66.65	364.21±66.65	496.16±72.28	590.83±35.31	531.06±100.28	524.59±22.87
7/2	404.25±56.96	404.25±56.96	378.46±42.11	617.07±56.05	429.07±66.95	461.29±70.15
7/9	894.92±73.35	894.92±73.35	932.51±158.9	793.1±16.98	474.78±94.72	741.03±62.51
7/16	1979.5±191.49	1979.5±191.49	622.75±74.27	1813.56±168.5	1762.44±278.05	1807.89±169.34
7/19	1666.46±253.92	1666.46±253.92	1776.17±219.46	1708.69±160.92	1617.48±346.2	1491.89±35.6
7/24	2146.32±190.05	2146.32±190.05	2079.05±228.13	1720.65±108.7	1540.16±251.56	1434.16±125.82
7/30	2693.6±256.38	2693.6±256.38	2441.31±390.61	2646.97±139.15	2113.37±165.72	2119.52±74.77
8/6	1958.03±134.61	1958.03±134.61	2529.99±364.46	2326.27±121.91	2169.33±239.66	1784.24±87.94
8/13	1963.09±97.65	1963.09±97.65	2007.61±116.93	1676.31±168.52	1186.72±337.46	1644.31±128.95
8/23	1346.09±262.34	1346.09±262.34	1528.26±17.05	1493.25±213.11	1316.24±320.36	1435.01±70.44
